# WebSpecmine: A Website for Metabolomics Data Analysis and Mining

**DOI:** 10.3390/metabo9100237

**Published:** 2019-10-19

**Authors:** Sara Cardoso, Telma Afonso, Marcelo Maraschin, Miguel Rocha

**Affiliations:** 1 CEB—Centre Biological Engineering, University of Minho, 4710-057 Braga, Portugal; saracardoso501@gmail.com (S.C.); telma.afonso94@gmail.com (T.A.); 2 Plant Morphogenesis and Biochemistry Laboratory, Federal University of Santa Catarina, Florianópolis SC 88040-900, Brazil; m.maraschin@ufsc.br

**Keywords:** metabolomics, statistical analysis, data mining, metabolite identification, pathway analysis, open-source software

## Abstract

Metabolomics data analysis is an important task in biomedical research. The available tools do not provide a wide variety of methods and data types, nor ways to store and share data and results generated. Thus, we have developed *WebSpecmine* to overcome the aforementioned limitations. *WebSpecmine* is a web-based application designed to perform the analysis of metabolomics data based on spectroscopic and chromatographic techniques (NMR, Infrared, UV-visible, and Raman, and LC/GC-MS) and compound concentrations. Users, even those not possessing programming skills, can access several analysis methods including univariate, unsupervised and supervised multivariate statistical analysis, as well as metabolite identification and pathway analysis, also being able to create accounts to store their data and results, either privately or publicly. The tool’s implementation is based in the R project, including its shiny web-based framework. *Webspecmine* is freely available, supporting all major browsers. We provide abundant documentation, including tutorials and a user guide with case studies.

## 1. Introduction

Omics technologies can characterise, in a global way, biomolecules and their interactions at a large scale. Metabolomics studies the whole (or part of a) metabolome, i.e., the set of the small molecules (<1000 Da) used as substrates or formed by the cell during biochemical reactions. As most of these reactions are enabled by enzymes, and since the metabolites they form or consume represent direct information about the cell’s metabolic activity, the measurement and analysis of the metabolome provide essential information to characterise a cell’s phenotype in response to genetic and environmental stimuli. Thus, there are many applications of metabolomics data analysis in fields as diverse as plant biology, nutrition, drug discovery and biomedicine, among others.

Mass Spectrometry, coupled with liquid or gas chromatography (LC/GC-MS), and Nuclear Magnetic Resonance (NMR) are the most used techniques to obtain relevant metabolomics data. However, Near and Mid Infrared (NIR and FTIR), Ultraviolet-Visible (UV-Vis), and Raman spectroscopies can also contribute with valuable data. These techniques are capable of generating large amounts of data, which asks for automated and efficient ways of conducting their analysis. Many tools have been put forward in recent years to address these tasks. However, most of them require programming skills and, if not, as is the case of web-based tools, they lack diversity in the available methods for pre-processing and analysing the dataset. In addition, they lack ways to freely store and share data and results in a user’s area that would allow a more convenient data analysis environment.

Therefore, developing an easy-to-use and freely accessible tool, suitable for users with no previous programming skills that can provide the analysis of a wide range of metabolomics data, by making use of diverse methods, is a relevant endeavour for the biological and biomedical fields. *WebSpecmine* was implemented so that the aforementioned shortcomings could be overcome, providing a user-friendly web-based application for loading, sharing, processing, analysing and mining metabolomics, providing state-of-the-art statistical and machine learning methods.

As an important advantage, in our tool, there is no fixed pipeline for data pre-processing and data analysis, providing extra flexibility when compared to previously developed tools, allowing for visualising and directly contrasting the results obtained following different approaches. In addition, we provide tools for the storage, pre-processing and analysis of spectroscopic data, such as NIR, FTIR, UV-Vis, and Raman, which none of the previously available applications supports.

## 2. Results

**Figure 1:**
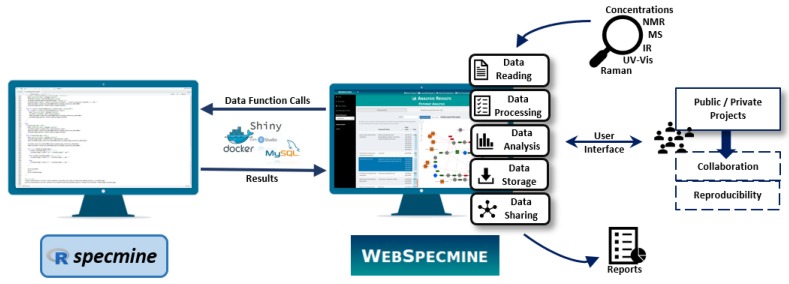
Global overview of *WebSpecmine*’s features and their implementation using the *specmine* R package and the tools *Shiny*, *MySQL* and *Docker*.

*WebSpecmine* is a user-friendly web-based application based on the R package *specmine* [1], previously developed by the authors’ research group. It is made freely available at [2]. A brief explanation on what this web-based tool has to offer is provided in the next sections. For more detailed information, the website provides abundant documentation (in the form of a Help page), including a user guide with a detailed description of the tool’s features and several tutorials with different case studies. Figure 1 gives an overview on how the website is implemented and its available features.

### 2.1. User Accounts

Although the main functionalities in the website are accessible without a user account, to share and store data and results, as well as to leave an analysis in ’stand-by’ and resume it later (saving it into a workspace containing data and results), a user account needs to be created. Data are stored into projects. A project is a study, or a group of studies, that contains data and metadata, as well as reports, tables and plots of the results obtained in their analysis. The user can choose if a given project should be private or public. If it is defined as public, everyone is able to see its contents and download the data files to their devices. However, only users that own an account can perform analysis over their data (a copy of the project is saved into the user account so that the content of the original project is not compromised). There are several public projects of published datasets, covering different types of data, made available by the website administrators, which are used, for instance, in the available tutorials. To create an account, the user has to send an email to the administrators of the site.

### 2.2. Loading and Visualising Data

**Figure 2:**
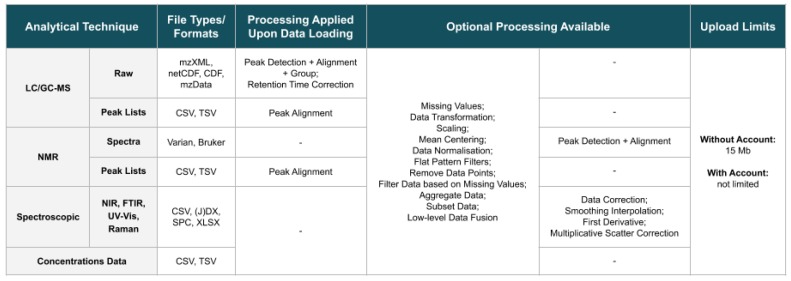
Summary of the file formats/types of the different analytical techniques supported, the processing methods performed on the respective data after data loading, further optional processing methods that can be performed at a later stage, and limits on the sizes of uploaded files.

The website supports raw LC and GC-MS spectra in mzXML, netCDF or mzData formats, while, for NMR, it supports FID signals in the Varian format and chemical shifts spectra in the Bruker format. By using *specmine*, Varian FID data are read using the Python module *NMRglue* [3], while Bruker files are read using functions from the *batman* R package [4]. Peak lists from these two techniques can also be submitted (in the CSV/TSV format). Regarding spectroscopic data, namely NIR, FTIR, UV-Vis, or Raman, the CSV, (J)DX, SPC and XLSX formats are supported. Reading (J)DX data are performed using the *ChemoSpec* R package [5], via *specmine*. For compound concentration data, i.e., concentration values defined for each quantified metabolite in each sample, the CSV/TSV format is supported. All data submitted can be accompanied by metadata information, stored in a CSV/TSV file. Figure 2 summarises the file formats/types of the analytical techniques supported by the website, as well as the processing performed on the different data types upon loading, and the limits on the sizes of the allowed files.

The process of loading data for analysis differs according to the type of user. Those not logged in will be asked to submit the data files from their devices; at the same time, a few options regarding the initial processing of the data and metadata files must be set. No data will be stored in the website, permanently or temporarily, except for MS raw data, temporarily stored while the user session is running. On the other hand, users with an account will be asked to select the data for analysis from the studies they have previously created in their accounts. Currently, the alignment of peaks for NMR and MS peak lists will be performed using an algorithm developed by the research group. LC/GC-MS raw data also undergo an initial stage of pre-processing after data loading, more specifically peak detection, peak alignment, and retention time correction. This MS raw data processing, performed via *specmine*, makes use of the loading and processing tools available in the XCMS tool [6,7].

In addition, for all users, available workspaces can be loaded. Users can also import NMR/ MS datasets from *MetaboLights* [8]. *WebSpecmine* makes available some of the public studies (according to the aforementioned data formats supported by the website) for users to copy to their accounts.

There are various ways to visualise loaded datasets. Besides showing the data and metadata tables, a brief statistical summary is shown, with detailed statistics for each sample and variable. Interactive boxplots can show the distribution of data variables, possibly conditional to the values of the samples for one or two metadata variables. In addition, an interactive plot shows peaks or spectral data. Reports with all information can be saved or downloaded.

### 2.3. Pre-Processing

Uploaded data can be processed using different methods organised in user-defined workflows (Figure 2). Different pipelines can be performed to compare results. After performing each pipeline, a new dataset is created. All datasets created, including the original one, are available to be analysed at any time during the session.

The set of available methods, for all types of data, includes: handling missing values, data transformations (e.g., log, cubic root), scaling, mean centering, data normalisation, and flat pattern filters. Users can also remove specified data points, or filter data based on the amount of missing values. Aggregating samples based on the metadata, creating subsets of data, and performing low-level data fusion with other datasets are also possible operations. Specifically for spectroscopic data, pre-processing methods also include data correction, smoothing interpolation, first derivative, and multiplicative scatter correction. Finally, the detection of peaks is available as a pre-processing method available for NMR spectral data. Most pre-processing methods implemented in *specmine*, and therefore used in our website, made use of other R packages, as it is detailed in the original paper for the interested reader [1].

### 2.4. Data Analysis

**Figure 3:**
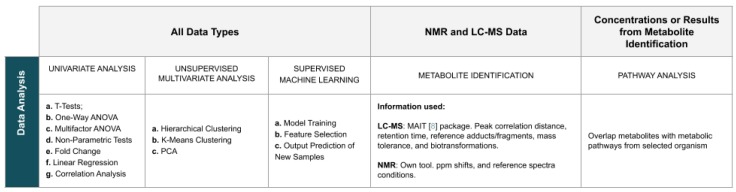
Summary of the *WebSpecmine*’s data analysis methods.

The application provides a wide variety of analysis methods, with options that can be personalised by the user. To simplify the search for methods, the page dedicated to data analysis is organised into eight panels: *Univariate Analysis*, *Principal Components Analysis (PCA)*, *Clustering Analysis*, *Machine Learning*, *Feature Selection*, *Metabolite Identification*, *Regression Analysis*, and *Pathway Analysis*. Figure 3 summarises the analysis methods available in the website.

Univariate statistical analysis methods such as t-tests, one-way and multifactor analysis of variance (ANOVA), non-parametric tests (Kruskal–Wallis and Kolmogorov–Smirnov), and fold change analysis are provided. Results consist of tables with numeric results and a *p*-value or fold change plot. Linear regression and correlation analyses provide results in the form of tables and plots.

Regarding unsupervised multivariate statistical analyses, clustering can be performed using K-means and hierarchical clustering. Results are shown as a dendrogram plot for the first, and a plot and a table with the assignment of samples into clusters for the latter. PCA can also be run, returning values for the components’ importance, a scores matrix and variable loadings, as well as a wide variety of plots, including a scree plot, a pairs plot, 2D/3D scores plots, and a biplot.

The spectrum of supervised machine learning models available include Partial Least Squares (PLS), Support Vector Machines (SVMs), Neural Networks (NN), Linear Discriminant Analysis (LDA), among others. Methods for model validation and optimisation of hyperparameters are available. For each trained model, the performance, the selected parameters, and the confusion matrix for the best combination of hyperparameters are returned. Performance values for each set of parameters tested are also provided, alongside with a table with the variables’ importance. For PLS models, a 3D plot is provided, as well as a plot and a table of the variables’ loadings. The prediction of new samples is available after performing model training, returning a table of the classes predicted for each sample. Feature selection methods include wrappers and filters. Results are shown accompanied by a performance plot, and a list of the variables composing the best performing subset.

Analyses that can provide added biological knowledge are also available. Metabolite identification for LC-MS and NMR data returns tables with the identified metabolites, including the identifier in the Human Metabolome Database (HMDB), and scores. LC-MS’s metabolite identification uses the *MAIT* R package [9], while for NMR a method developed by the authors and not yet published is used. Pathway analysis for compound concentrations, or for data obtained from the metabolite identification, provides a table with the information on the pathways identified and an interactive pathway map that shows each pathway present in the table, a task also implemented by the authors. Globally, tables with results can be downloaded by the users for further detailed analysis, as well as automatically generated reports in several formats, including HTML and PDF.

### 2.5. Application of *WebSpecmine* to a Case Study

To demonstrate the aforementioned features of *WebSpecmine*, we reproduced the analysis of a metabolomics study. Thus, we are able to show the utility of this website in performing the same tasks executed by other studies, but in a simpler and faster manner, without the need for programming skills. The reproduction of the case study is explained in detail in the Supplementary Materials. A workspace (*Cassava PPD : IR Data (DX files)*) with all the datasets and results generated was saved and is publicly available in the website.

## 3. Discussion

The website presented here allows the visualisation, processing, and analysis of several types of metabolomics data in a flexible and user-friendly manner. One important advantage is the flexibility of creating different pipelines both for analysis and pre-processing, allowing for comparing their results. For instance, the same pre-processing tasks performed in a different order can affect the final processed dataset, potentially leading to different results in the analysis. In our tool, users can perform any set of pre-processing methods in the desired order, and create as many processing pipelines over the same data as needed, so that posterior results can be compared. Furthermore, once the data are loaded and pre-processed, any analysis applicable to that specific type of data is available to be performed, without the need to reload the data every time one wants to perform a new analysis. Indeed, all results and datasets generated in the current session are easily accessible in the sidebar panel of the website.

In fact, such a flexible way of handling data, processing and analysis is not accomplished in *MetaboAnalyst* [10], one of the most remarkable available web-tools for metabolomics data that instead has a fixed processing pipeline, and makes the user upload data every time a new type of analysis needs to be performed. While *MetaboAnalyst* provides a fixed pipeline for processing data, where the order in which the different processing methods are applied cannot be changed by the users, which include treatment of missing values, data filtering, normalisation, transformation and scaling, *WebSpecmine* not only allows users to perform their processing pipeline in the desired order, but also provides further processing methods. Furthermore, *MetaboAnalyst* does not have an area where users can store data and results to be accessed later, forcing them to download results files before they leave. Regarding analysis methods, *MetaboAnalyst* does not have a diverse set of machine learning models, does not perform metabolite identification, or support any type of spectroscopic data. However, *MetaboAnalyst* provides methods not yet covered by *WebSpecmine*, which include biomarker, time-series, and enrichment analysis.

Another web-tool worth mentioning is *XCMSonline* [7], a tool more robust at processing MS raw data, the pipeline implemented in our website being the one provided by the R version of this tool. As *XCMSonline* is mainly a processing tool, *WebSpecmine* implements statistical analyses beyond those provided by *XCMSonline*, as well as other types of analysis, such as metabolite identification and pathway analysis. Furthermore, *XCMSonline* does not support metabolomics data other than MS and it only provides its tools in fixed pipelines. In addition, although it allows users to share their data, one must create an account to use this tool.

Galaxy workflows such as *Galaxy-M* [11], *Workflow4Metabolomics* [12] and *PhenoMeNal* [13] are also a very interesting platform to combine different methods and perform analysis of metabolomics data. However, users may have to take some time to get used to and learn how to work in the Galaxy environment, as it may not be completely straightforward for all. In addition, some workflows require a user login to use the tool. Furthermore, unlike Galaxy tools, *WebSpecmine* users do not need to set a priori a workflow to be run, but instead can decide the different pre-processing pipelines and analyses to make over the processed data along the way. Indeed, the intuitive way results are shown allows users to choose the next analysis step in their workflow based on the results they have obtained thus far.

Furthermore, none of the web-tools mentioned above performs data analysis of spectral data, a feature of *WebSpecmine*.

Nevertheless, we do recognise that *WebSpecmine* has its own limitations, especially regarding analysis methods not yet covered in our website, or even storage capacity and analysis of large sets of data. To overcome this, we made an installable version of the website available, so that groups with more powerful servers can install and use it locally. Our aim is to improve our website with additional tools, providing users with methods as comprehensive as possible, in a single place.

## 4. Materials and Methods

### 4.1. Website Implementation

The web-based application makes use of the functions provided by the *specmine* package [1], previously developed by the research group for the R environment. It allows for performing analysis of metabolomics or spectroscopic data, as well as compound concentration datasets. The R package *shiny* [14] was used to develop our website, building an interactive web application. MySQL was used to create and manage the database that ensures data persistence and user profiles. To put together the website on the server, the docker compose tool was used, easing the configuration of new instances. In our available instance, the docker is currently installed on a server with two Intel Xeon X5650 processors (Santa Clara, CA, USA) and RAM 64GB ECC DDR3, although this server should be improved in a near future.

For users with no account, a maximum of 15 Mb was set for the size of the uploaded data files, while account users do not have any limitations for now. Regarding loading and initial processing times, these vary depending on the data type, as they require different processing methods. MS and NMR spectra data are those that spend more time due to the processing required and data formats, with times spanning from approximately 2 s for a concentration dataset of around 30 kB in size, to approximately 5 min for an NMR spectra dataset of around 18 MB in size.

### 4.2. Desktop Version

Due to the limited computational resources, the website has limitations on its capacity to store or analyse large sets of data. Therefore, we make available an installable version of the website, so that groups with more powerful servers can install and use it locally. For this purpose, we have put together a docker, easing its installation. All functionalities available in the website are also available, including the database where different users are allowed to register, if the maintainer of the local application chooses to do so. However, any data stored in our website, public or private, will not be made available. The desktop version is available in https://gitlab.bio.di.uminho.pt/WebSpecmine/desktop_docker, further allowing to check the source code.

## 5. Conclusions

We created a powerful web application that allows extensive analysis of various types of metabolomics data, where users can also share their data and results with the community. The application is available in our webserver, but can also be installed in other locations, since the source code is made available for the community. This will allow groups with large datasets to install the application over more powerful servers, since our computational resources are limited. Furthermore, our website will be constantly improved and updated regarding community’s achievements.

## Supplementary Materials

The following are available at https://www.mdpi.com/2218-1989/9/10/237/s1, supplementary file: Details of the reproduction of the case study.

## Abbreviations

The following abbreviations are used in this manuscript:

ANOVA	Analysis of Variance
CSV	Comma Separated Values
FID	Free Induction Decay
FTIR	Fourier-Transform Infrared
GC	Gas Chromatography
HMDB	Human Metabolome Database
LC	Liquid Chromatography
LDA	Linear Discriminant Analysis
MS	Mass Spectrometry
NIR	Near Infrared
NMR	Nuclear Magnetic Resonance
NN	Neural Networks
PCA	Principal Component Analysis
PLS	Partial Least Squares
SVMs	Support Vector Machines
TSV	Tab Separated Values
UV	Ultra-Violet
