# Supplementary material for: WebSpecmine: A Website for Metabolomics Data Analysis and Mining
Source: Metabolites. 2019 Oct 19;9(10):237. doi: 10.3390/metabo9100237 (PMC6835413; doi:10.3390/metabo9100237)
Supplement: Supplementary file 1 [file metabolites-09-00237-s001.pdf]

Supplementary Material

Application of *WebSpecmine* to a case study

The selected case study relates to cassava roots' postharvest physiological deterioration (PPD) over 5 different time periods [1]. The studied stages of deterioration consisted on 0 (fresh samples), 3, 5, and 8 days post harvesting. The cassava roots are well known for their important source for dietary and industrial carbohydrates, although, as a tropical root crop, it is a postharvest deterioration sensitive species, caused by either physiological or microbiological processes. A total of 80 samples were collected using the Fourier-transform infrared spectroscopy (IR) technique, with a spectral window from 4000 to 400 cm^{-1}, at the specified stages of PPD and for four different cultivars, namely Branco (BRA), IAC576-70 (IAC), 183 Oriental (ORI), and SCS253 Sangão (SAN). The collected samples were produced over the growing season of 2011/2012.

Data regarding each of these samples were stored in DX files, where each corresponds to a FTIR spectrum. Information regarding two metadata variables considered, named *cultivars* and *ppds* (stage) in the data files, was stored in a CSV file. Data files were supplied by the authors and made available at the *WebSpecmine* app, under the data folder *IR Data (DX files)*, as the public project *Cassava PPD*. In the same project, the metadata file was stored at the metadata folder under the name *metadata.csv.* To reproduce this study, users have to import the project from the *Public Projects* page, accessible through the sidebar panel, to their personal account (Supplementary Figure S1).

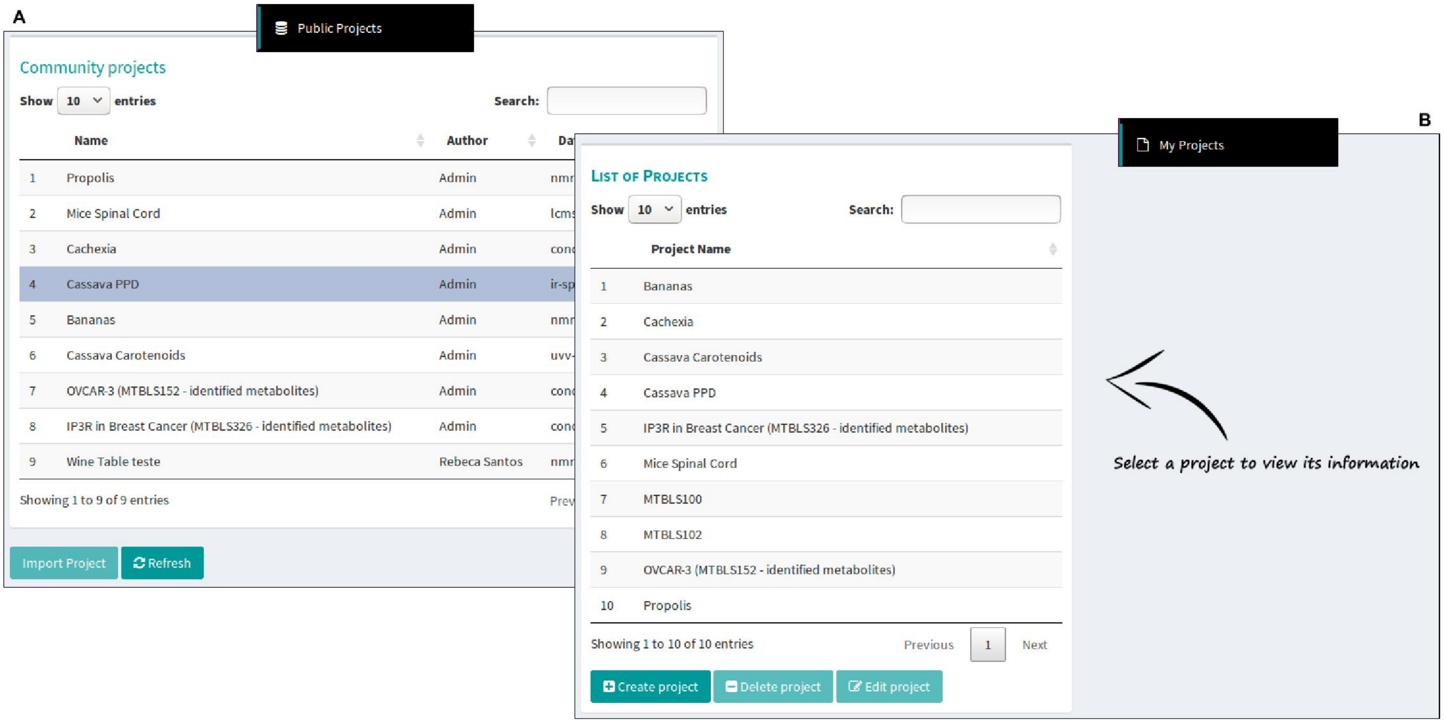

Figure S1: Illustration of how to import the public project *Cassava PPD*. (A) In the *Public Projects* page, accessed through the sidebar panel by clicking the tab with the same name, the user selects the *Cassava PPD* project in the table of projects and clicks the *Import Project* button. (B) By accessing the user's personal space, by clicking the tab *My Projects* present in the sidebar panel, the user can confirm that the imported project has actually been copied.

To select the wanted data for analysis (Supplementary Figure S2), the user clicks the *Choose Files* button. A pop-up window appears, so that the project and respective data folder and metadata file can be selected. After that, data and metadata options can be set, before finalizing the submission of the files for analysis. Once the dataset is loaded into the session, it is named by default as *OriginalData*. All datasets created during the current session are stored.

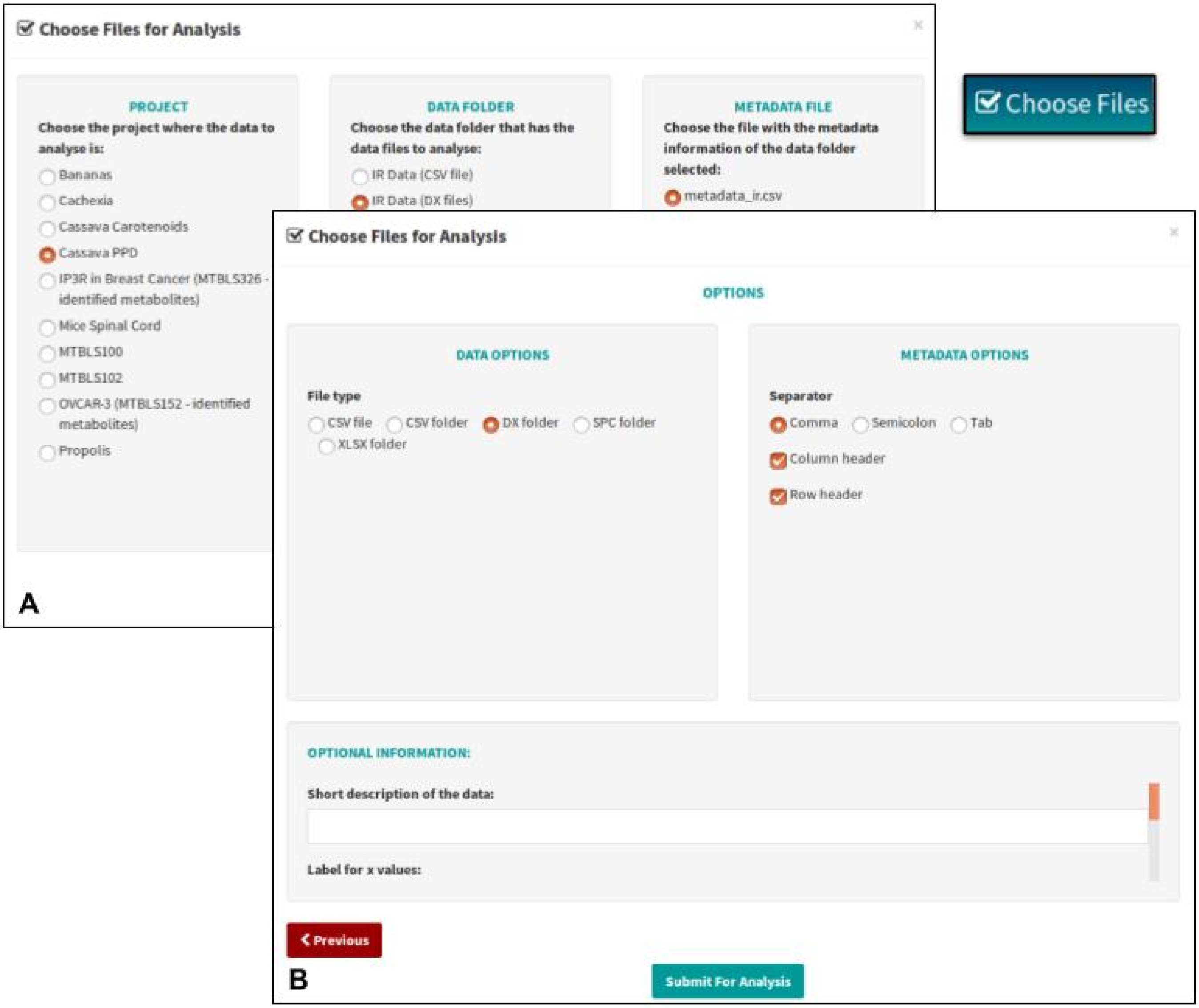

Figure S2: Illustration of how to choose the correct files, once the *Cassava PPD* project is copied to the user account. After clicking the *Choose Files* button in the header panel, the user will see (A) a pop-up window where the project (*Cassava PPD*), the correct data folder from this project (*IR Data (DX files)*) and the metadata file (*metadata_ir.csv*) are chosen. After clicking *Next*, (B) the data and metadata options must be set to correctly read the files. After setting these options, as shown in the figure, the *Submit For Analysis* button can be clicked, so that the dataset is created and uploaded into the session.

Before starting the analysis, pre-processing was performed (Supplementary Figure S3). For this, the *Pre-Processing* page was accessed through the header panel. Smoothing interpolation using the Bin method, with a reducing factor of 10, was applied, followed by converting the numerical values of the PPD metadata variable (*ppds*) into factors, so that classification can be correctly performed later on. The new dataset was named *cassava_processed*. After clicking *Finish*, the new dataset is made available in the *Dataset being used* section.

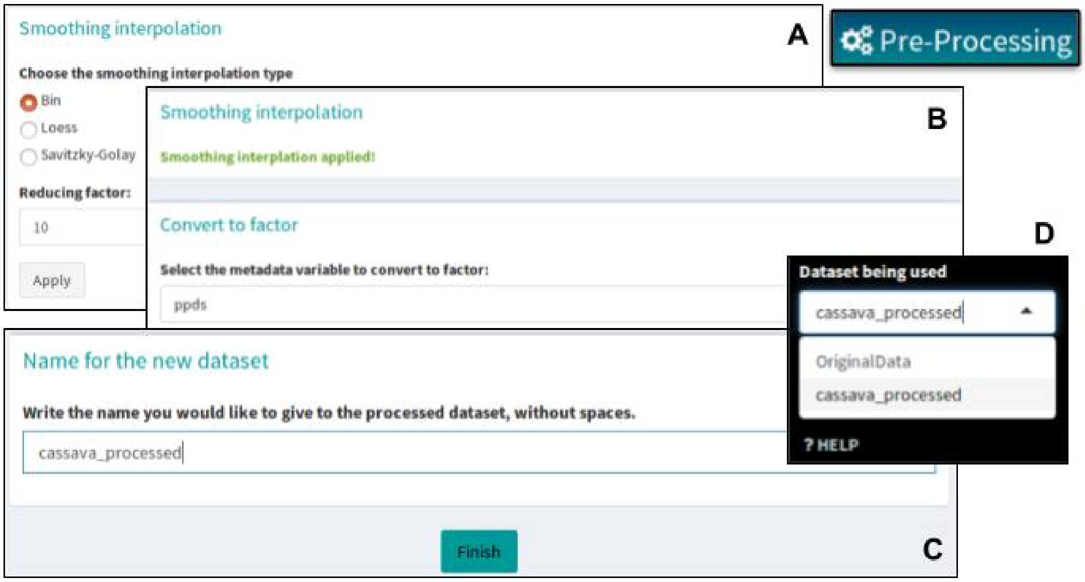

Figure S3: Illustration of how to pre-process the dataset, once it is uploaded into the session. After clicking the *Pre-Processing* button in the header panel, the user will have to (A) find the *Smoothing interpolation* box in this page, set the options and click *Apply*. Then, (B) in the *Convert to factor* box, the metadata variable *ppds*, representing the PPD of the samples, should be selected to convert its values into factors (nominal variable). To save the processing pipeline as a new dataset, (C) the name for this new dataset must be given in the *Name for the new dataset* box, followed by clicking the button *Finish*, present at the end of the page. (D) The new dataset will be available at the *Dataset being used* section at the sidebar panel.

To visualize the effect of smoothing interpolation in the spectral data (supplementary figures S4), the *OriginalData* dataset can be selected (at the *Dataset being used* section) and the *Data Visualization* page may be accessed, through the header panel, to observe the spectra plot, followed by selecting the new dataset *cassava_processed*, which will allow to see the respective plot.

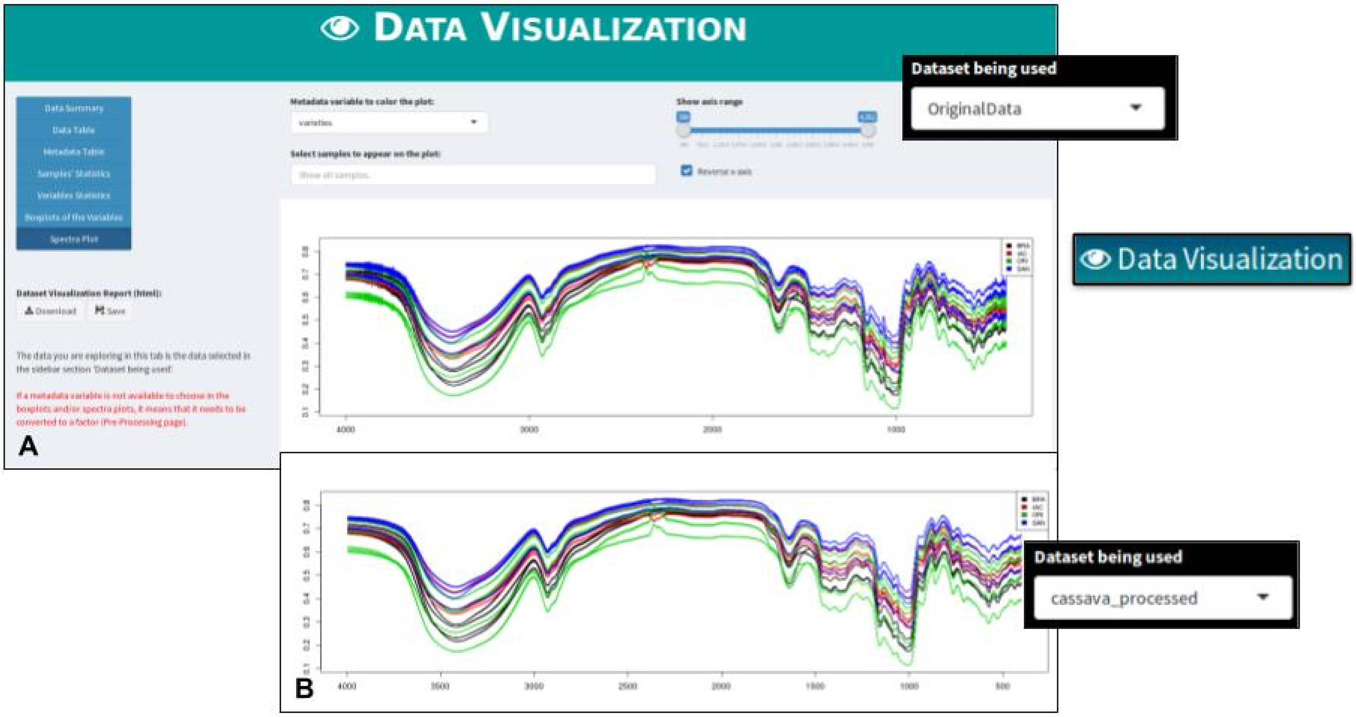

Figure S4: To visualize the effect of smoothing interpolation on the spectral data, the user can (A) select the *Data Visualization* button at the header panel after selecting the *OriginalData* dataset and select the *Spectra Plot* option at the left of the page. Then, (B) by selecting the *cassava_processed* dataset, the spectra plot that will appear will correspond to this dataset.

The authors started by conducting a Principal Components Analysis (PCA) on the dataset, with the intention of evaluating the most important biochemical events related to the deterioration changes and to discriminate cassava cultivars during PPD.

As the authors only used the data in the spectra range of 3000 to 600 cm^{-1}, having the *cassava_processed* dataset selected, we went back to the *Pre-Processing* page and created a subset of the dataset by selecting the interval of values we wanted to retain (from 3000 to 600 cm^{-1}), as in Supplementary Figure S6. The new dataset was named *cassavaProc_600_3000*.

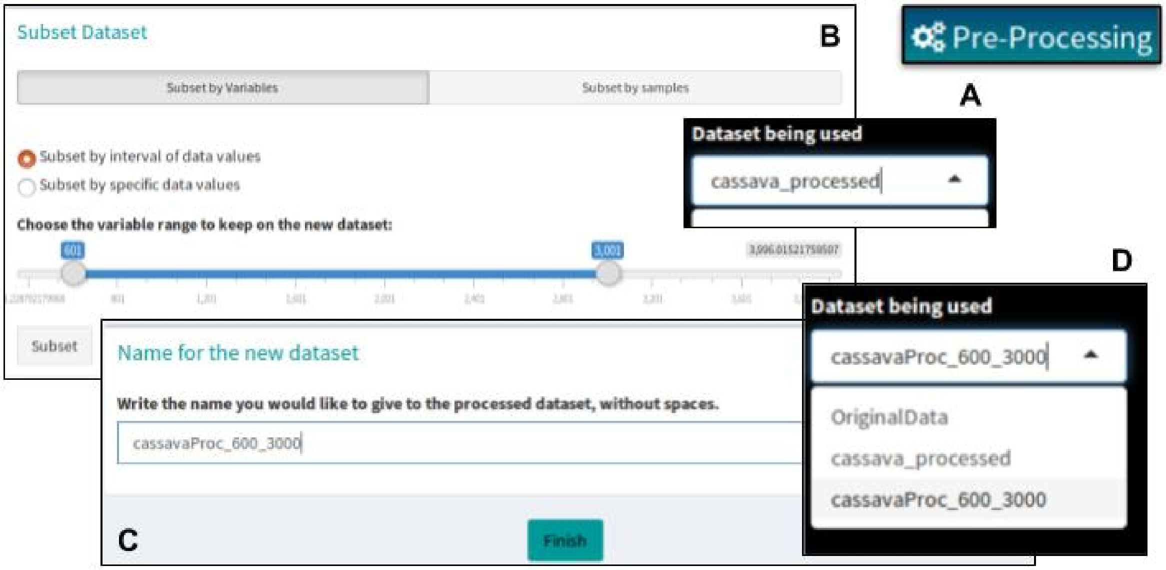

Figure S6: Illustration of how-to subset the dataset to only retain the data points in the spectral width of 3000 – 600 cm^{-1}. (A) Having the *cassava_processed* dataset selected, the user can go back to the Pre-Processing page and, (B) in the *Subset Dataset* box, subset the dataset by selecting the variable range in question. To save the processing pipeline in a new dataset, (C) the name for this new dataset must be given in the *Name for the new dataset* box, followed by clicking the button *Finish*, present at the end of the page. (D) The new dataset will be available at the *Dataset being used* section at the sidebar panel.

Having the *cassavaProc_600_3000* dataset selected, PCA was performed, by entering the *Principal Components Analysis (PCA)* box in the *Run Analysis* page, accessed through the header panel. The dataset was centred prior to PCA analysis, set in the options for this method (Supplementary Figure S7). The analysis was named *PCA_3000_600*. After being redirected to the results page of this PCA analysis, all numerical results available may be checked and personalized plots may be generated (Supplementary Figure S7). A two-dimensional plot of the two first components was generated by accessing the *Scores Plot 2D* tab in the *Make plots* section, by colouring the data points using the PPD metadata values.

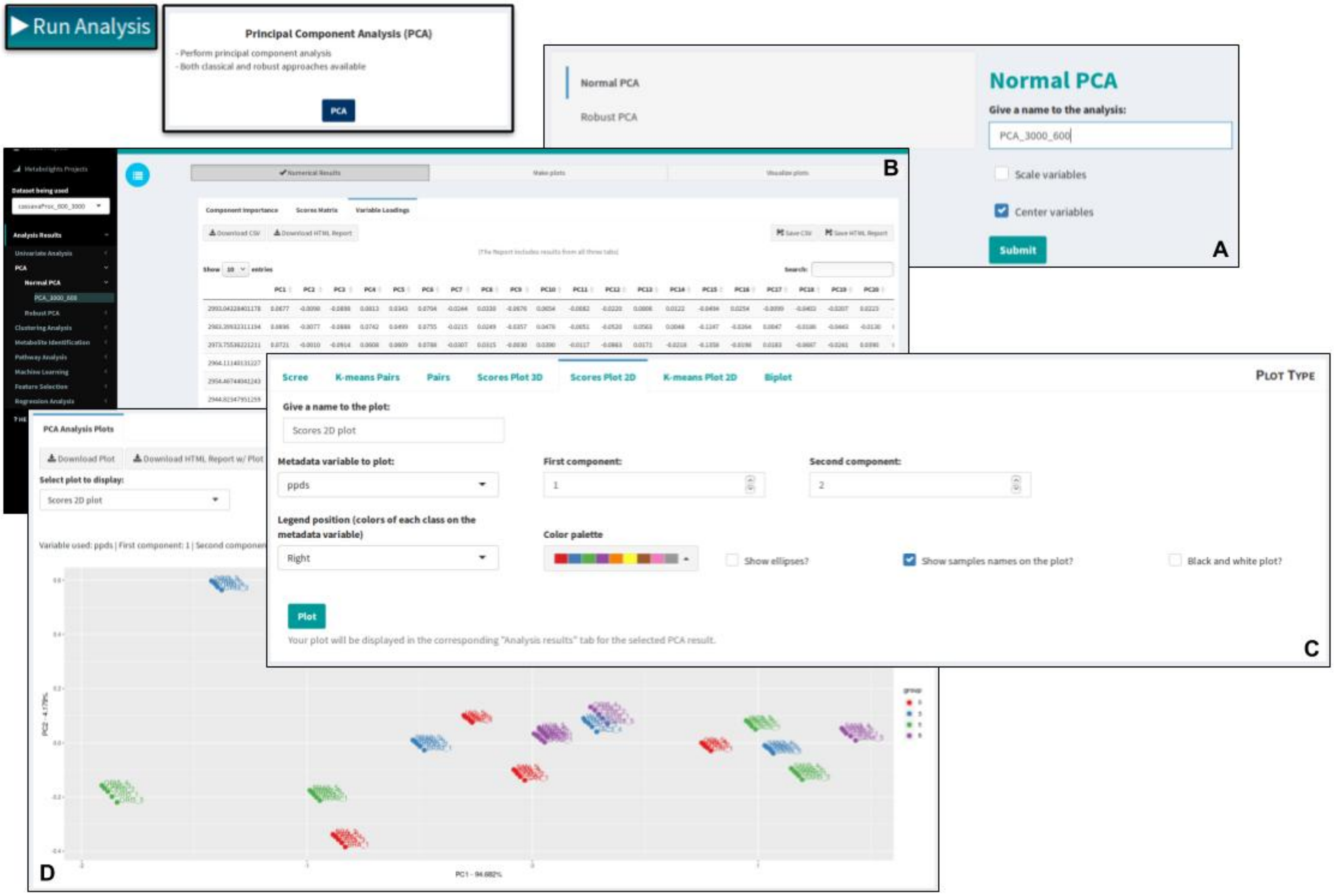

Figure S7: Illustration of how to perform the PCA and obtain the two-dimensional scores plot from the first and second PCs. After selecting the *cassavaProc_600_3000* dataset, the user clicks the button *Run Analysis* at the header panel. In the *Run Analysis* page, the *Principal Components Analysis (PCA)* box should be selected. After that, (A) PCA is performed, by ticking the option to centre the variables prior to the analysis and giving a name to the analysis. Once the user clicks *Submit*, the analysis starts and, when finished, (B) the site redirects to the results page. Here, (C) the user can go to the *Make plots* section, select the *Score Plot 2D* tab and set the option to personalize the results plot. Then, the button *Plot* should be clicked, so that the plot is created and (D) shown in the *Visualize Plots* section.

Some interesting conclusions can be deduced (Supplementary Figure S8). For example, there was a clear separation between the varieties BRA and ORI, which are the susceptible and tolerant to PPD genotypes, respectively. On the other hand, the total variance explained only by the first component is of approximately 95%.

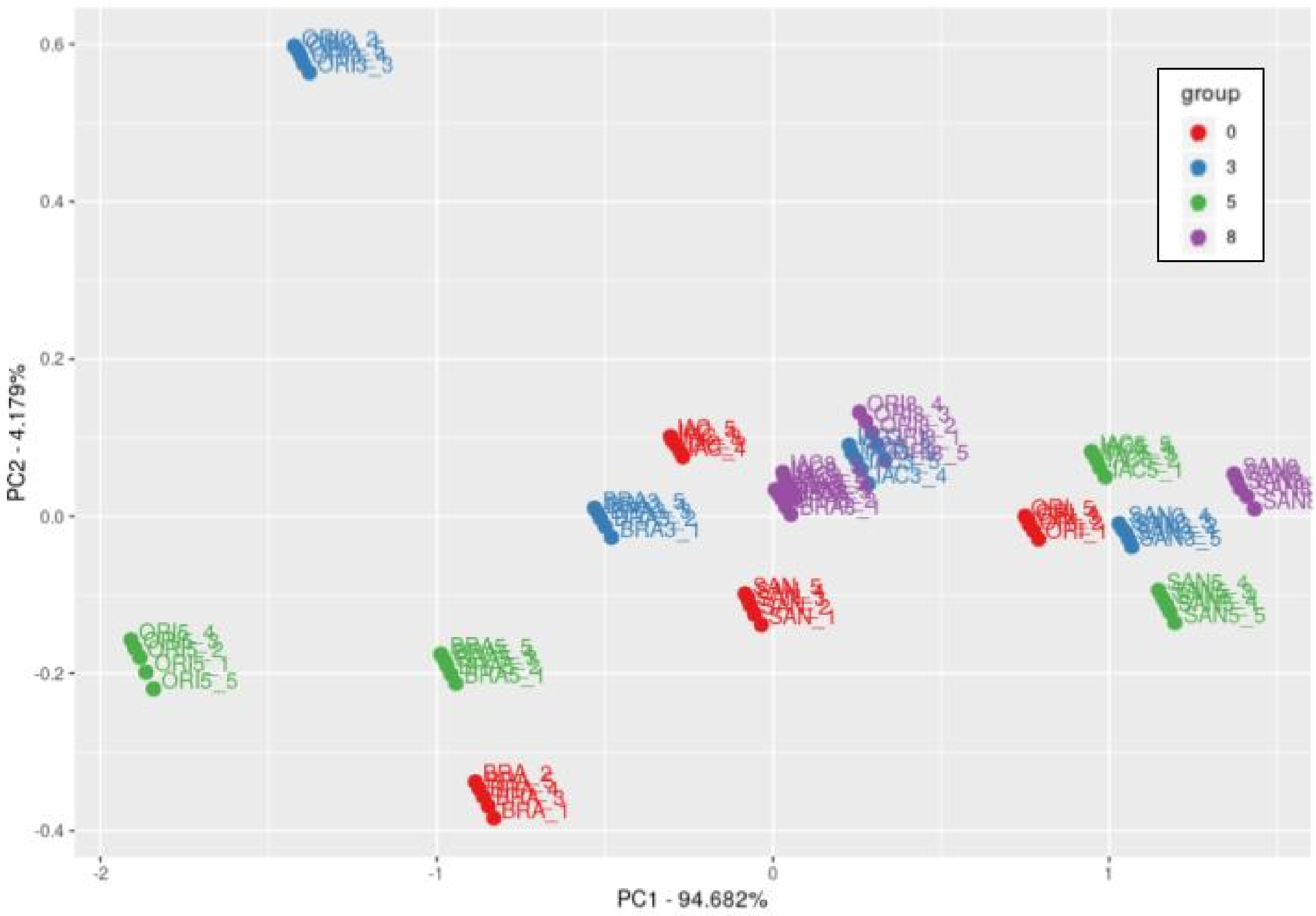

Figure S8: Scores plots of the first and second components of PCA on the spectra region from 3000 to 600 cm^{-1}, obtained by *WebSpecmine*.

We further separated the dataset into three regions, as mentioned in the original article, typical of carbohydrates (1200-900 cm^{-1}), proteins (1680-1000 cm^{-1}), and lipids (3000-1700 cm^{-1}), to see if any of these regions could discriminate the cultivars according to their biochemical discrepancies over the PPD. For this, the same process for the analysis of PCA was repeated three times, after creating three new datasets, by subsetting the *cassavaProc_600_3000* in each of the three different spectral regions mentioned. These datasets were named *cassavaProc_carbohyd*, *cassavaProc_proteins*, *cassavaProc_lipids*, respectively, with the respective data analysis being named as *PCA_carbohydrates*, *PCA_proteins* and *PCA_lipids*.

In fact, we also observed that carbohydrates and proteins were the best ones at performing this screening.

In the original study, the authors trained several classification models, including SVMs and Decision Trees, to test the ability to discriminate deterioration stages (PPD) along different groups of cultivars.

After re-selecting the *cassavaProc_600_3000* dataset, we entered the *Machine Learning* box in the *Run Analysis* page. Parameter optimization was performed by testing 10 different values for each models' parameters, while cross validation with 10 folds and accuracy as the performance metric were chosen (Supplementary Figure S9). The analysis was named *models_PPDS*.

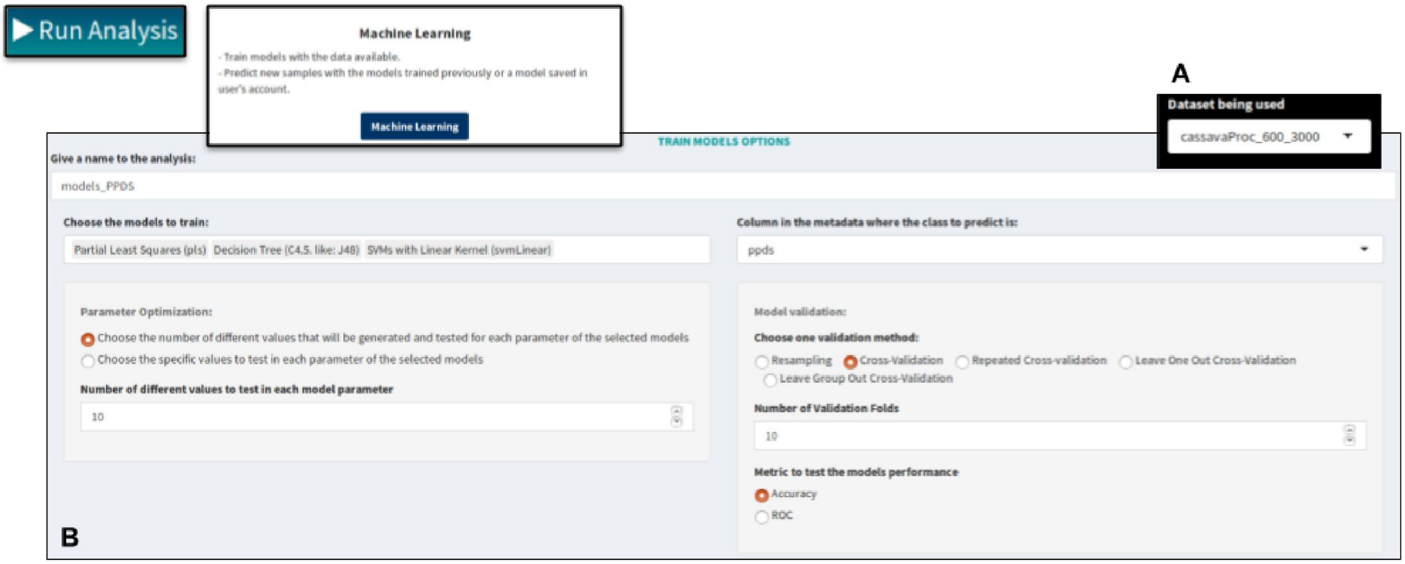

Figure S9: Illustration of how to perform model training of PLS, SVM and Decision Tree models. (A) Having the *cassavaProc_600_3000* dataset selected, the user should access the *Machine Learning* box in the *Run Analysis* page. (B) Once here, the user can choose the models to train (PLS, Decision Tree and SVM), and set the *Parameter Optimization* and *Model Validation* options as shown. A name to the analysis should also be provided. Once all is set, the user may click the button *Train Models*, at the bottom of the page, to start the analysis. Once the models are trained, the site will redirect to the respective results page.

After being redirected to the results page of this model training analysis, the results can be visualized (Supplementary Figure S10), with special emphasis on the confusion matrices obtained and the accuracy results of each model according to the different hyperparameters tested.

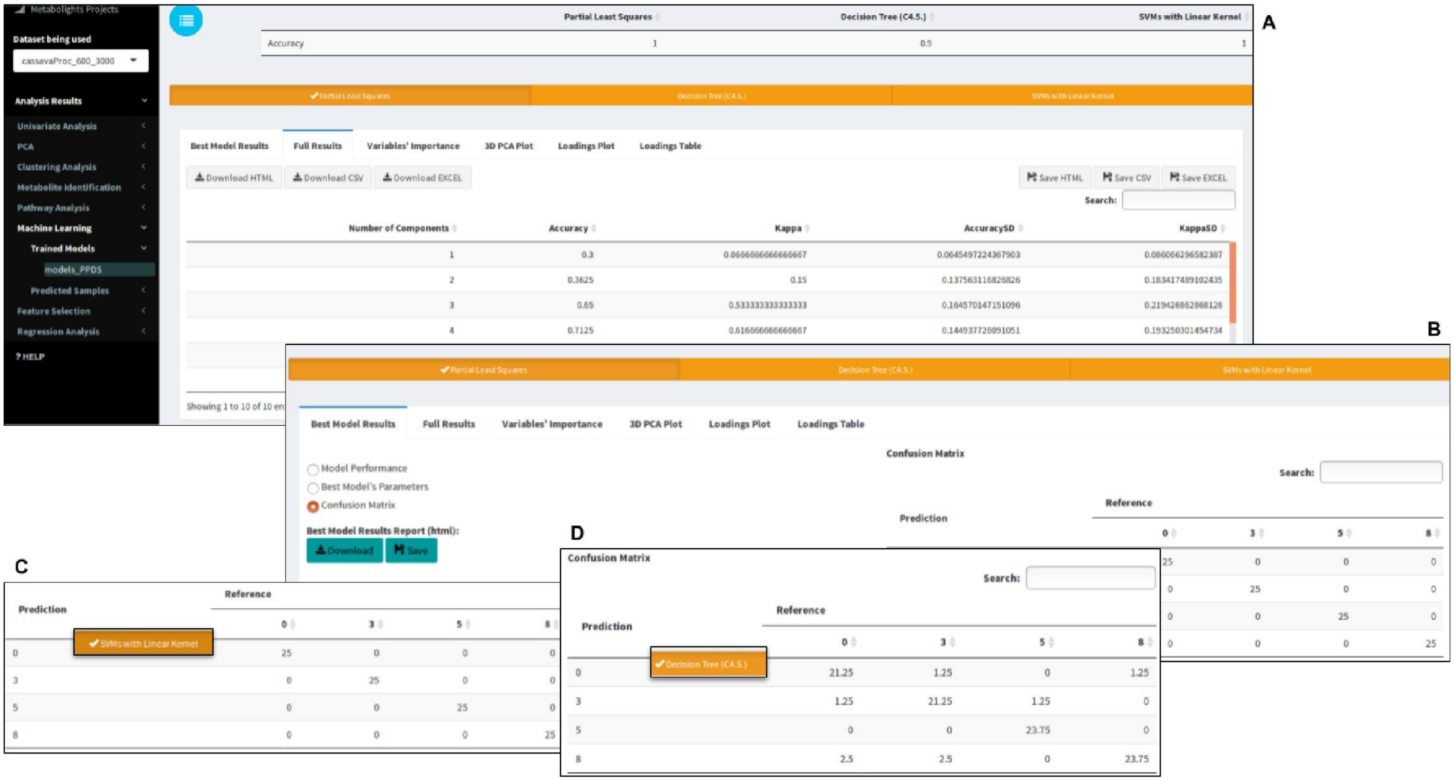

Figure S10: Images of the results that can be found in the page of the models trained. (A) In this page, the user is able to see a summary table with the accuracies of each trained model. Furthermore, there is a section reserved for each model trained, where one can find the respective results. Here, it is given special emphasis to the confusion matrices, accessible through the *Best Model Results* tab for each model: (B) Partial Least Squares, (C) SVMs with Linear Kernel and (D) Decision Tree.

The main conclusion drawn by the authors from such analysis consisted on the fact that the SVM model showed the best performance at clearly separating the samples from different deterioration stages (PPD) across the different cultivars. The same could be accomplished with our results.

A hierarchical clustering on samples was performed next, with the objective of assessing the similarity within samples. They concluded that four clear clusters emerged, although not clearly by either cultivars or PPD. The same results could be drawn by our analysis (Supplementary Figure S11).

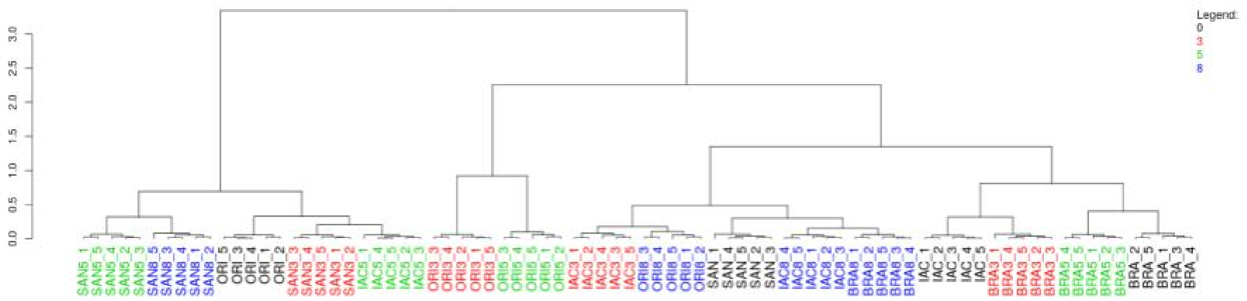

Figure S11: Dendrogram plot from the hierarchical clustering analysis performed on *WebSpecmine*.

Hierarchical Clustering on samples was performed by entering the *Clustering Analysis* box in the *Run Analysis* page. The *cassavaProc_600_3000* dataset was selected to perform this analysis. As mentioned by the authors, the hierarchical clustering was performed with an Euclidean distance and a complete aggregation method (Supplementary Figure S12). The analysis was named *HC_600_3000*. After being redirected to the results of Clustering Analysis, the numerical results can be checked and the user can personalize a dendrogram plot (Supplementary Figure S12).

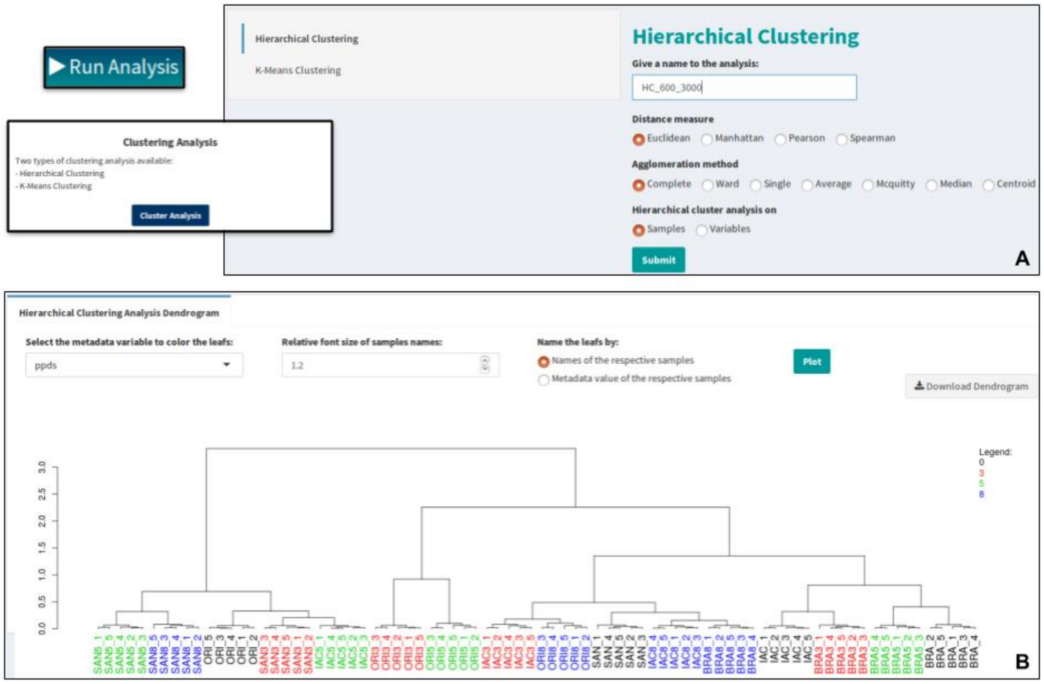

Figure S12: Illustration of how to perform the Hierarchical Clustering and obtain the dendrogram plot of the results. After selecting the *cassavaProc_600_3000* dataset, the user clicks the button *Run Analysis* at the header panel. Once in the *Run Analysis* page, the *Clustering Analysis* box should be selected. After that, (A) *Hierarchical Clustering* is performed, by setting the desired options and naming the analysis. Once the user clicks *Submit*, the analysis starts and, when finished, (B) the site redirects to the results page. Here, the user can go to the *Dendrogram* section and personalize the plot. Here, the samples in the plot were represented by their respective names and coloured by the deterioration stage (PPD).

A workspace with all these datasets and results (*Cassava PPD : IR Data (DX files)*) was saved and is publicly available.

Overall, we could replicate the evaluated study. Thus, we here showed how the website can be very useful to rapidly and efficiently analyse metabolomics data in a simple way, without the user having the need to write any program or script.

References

[1] Uarrota, V.G., Moresco, R., Coelho, B., Costa Nunes, E., Peruch, L.A.M., Oliveira Neubert, E., Rocha, M., & Maraschin, M. (2014) Metabolomics combined with chemometric tools (PCA, HCA, PLS-DA and SVM) for screening cassava (Manihot esculenta Crantz) roots during postharvest physiological deterioration. *Food Chemistry*, 161, 67–78.
